# Supplementary material for: Pyruvate Dehydrogenase Kinase 1 inhibition mediated oxidative phosphorylation enhancement in cartilage promotes osteoarthritis progression
Source: BMC Musculoskelet Disord. 2023 Jul 20;24:597. doi: 10.1186/s12891-023-06585-6 (PMC10357736; doi:10.1186/s12891-023-06585-6)
Supplement: Supplementary file 1 — Additional file 1. [file 12891_2023_6585_MOESM1_ESM.zip › BP.Gsea.1653623667859/GOBP_NUCLEOSIDE_BISPHOSPHATE_BIOSYNTHETIC_PROCESS.html]

Details for gene set GOBP\_NUCLEOSIDE\_BISPHOSPHATE\_BIOSYNTHETIC\_PROCESS[GSEA]

|  || Dataset | 55457\_collapsed\_to\_symbols.cls.cls #OA\_versus\_normal.cls.cls #OA\_versus\_normal\_repos |
| Phenotype | cls.cls#OA\_versus\_normal\_repos |
| Upregulated in class | OA |
| GeneSet | GOBP\_NUCLEOSIDE\_BISPHOSPHATE\_BIOSYNTHETIC\_PROCESS |
| Enrichment Score (ES) | 0.378559 |
| Normalized Enrichment Score (NES) | 1.566036 |
| Nominal p-value | 0.014888338 |
| FDR q-value | 0.2897489 |
| FWER p-Value | 1.0 |
Table: GSEA Results Summary

  

Fig 1: Enrichment plot: GOBP\_NUCLEOSIDE\_BISPHOSPHATE\_BIOSYNTHETIC\_PROCESS      
 Profile of the Running ES Score & Positions of GeneSet Members on the Rank Ordered List

  

| SYMBOL | TITLE | RANK IN GENE LIST | RANK METRIC SCORE | RUNNING ES | CORE ENRICHMENT || 1 | MPC2 | mitochondrial pyruvate carrier 2 [Source:HGNC Symbol;Acc:HGNC:24515] | 140 | 0.870 | 0.0388 | Yes |
| 2 | PDHB | pyruvate dehydrogenase E1 subunit beta [Source:HGNC Symbol;Acc:HGNC:8808] | 149 | 0.862 | 0.0875 | Yes |
| 3 | PANK2 | pantothenate kinase 2 [Source:HGNC Symbol;Acc:HGNC:15894] | 220 | 0.805 | 0.1281 | Yes |
| 4 | DLD | dihydrolipoamide dehydrogenase [Source:HGNC Symbol;Acc:HGNC:2898] | 565 | 0.635 | 0.1376 | Yes |
| 5 | PAPSS2 | 3'-phosphoadenosine 5'-phosphosulfate synthase 2 [Source:HGNC Symbol;Acc:HGNC:8604] | 592 | 0.627 | 0.1714 | Yes |
| 6 | PAPSS1 | 3'-phosphoadenosine 5'-phosphosulfate synthase 1 [Source:HGNC Symbol;Acc:HGNC:8603] | 703 | 0.593 | 0.1967 | Yes |
| 7 | PPCS | phosphopantothenoylcysteine synthetase [Source:HGNC Symbol;Acc:HGNC:25686] | 716 | 0.589 | 0.2295 | Yes |
| 8 | HSD17B12 | hydroxysteroid 17-beta dehydrogenase 12 [Source:HGNC Symbol;Acc:HGNC:18646] | 795 | 0.570 | 0.2560 | Yes |
| 9 | ACOT7 | acyl-CoA thioesterase 7 [Source:HGNC Symbol;Acc:HGNC:24157] | 971 | 0.524 | 0.2724 | Yes |
| 10 | ACAT1 | acetyl-CoA acetyltransferase 1 [Source:HGNC Symbol;Acc:HGNC:93] | 1017 | 0.513 | 0.2982 | Yes |
| 11 | PPT1 | palmitoyl-protein thioesterase 1 [Source:HGNC Symbol;Acc:HGNC:9325] | 1042 | 0.508 | 0.3254 | Yes |
| 12 | TECR | "trans-2,3-enoyl-CoA reductase [Source:HGNC Symbol;Acc:HGNC:4551]" | 1047 | 0.508 | 0.3542 | Yes |
| 13 | PDK2 | pyruvate dehydrogenase kinase 2 [Source:HGNC Symbol;Acc:HGNC:8810] | 1772 | 0.388 | 0.3200 | Yes |
| 14 | SNCA | synuclein alpha [Source:HGNC Symbol;Acc:HGNC:11138] | 1800 | 0.384 | 0.3399 | Yes |
| 15 | ELOVL4 | ELOVL fatty acid elongase 4 [Source:HGNC Symbol;Acc:HGNC:14415] | 2017 | 0.360 | 0.3436 | Yes |
| 16 | PDK1 | pyruvate dehydrogenase kinase 1 [Source:HGNC Symbol;Acc:HGNC:8809] | 2124 | 0.347 | 0.3552 | Yes |
| 17 | PPCDC | phosphopantothenoylcysteine decarboxylase [Source:HGNC Symbol;Acc:HGNC:28107] | 2251 | 0.332 | 0.3644 | Yes |
| 18 | PDHA1 | pyruvate dehydrogenase E1 subunit alpha 1 [Source:HGNC Symbol;Acc:HGNC:8806] | 2433 | 0.310 | 0.3680 | Yes |
| 19 | ACSBG1 | acyl-CoA synthetase bubblegum family member 1 [Source:HGNC Symbol;Acc:HGNC:29567] | 2520 | 0.301 | 0.3786 | Yes |
| 20 | ELOVL6 | ELOVL fatty acid elongase 6 [Source:HGNC Symbol;Acc:HGNC:15829] | 3253 | 0.226 | 0.3345 | No |
| 21 | COASY | Coenzyme A synthase [Source:HGNC Symbol;Acc:HGNC:29932] | 3498 | 0.202 | 0.3271 | No |
| 22 | MLYCD | malonyl-CoA decarboxylase [Source:HGNC Symbol;Acc:HGNC:7150] | 4852 | 0.083 | 0.2265 | No |
| 23 | HACD1 | 3-hydroxyacyl-CoA dehydratase 1 [Source:HGNC Symbol;Acc:HGNC:9639] | 5342 | 0.044 | 0.1910 | No |
| 24 | PANK4 | pantothenate kinase 4 (inactive) [Source:HGNC Symbol;Acc:HGNC:19366] | 5382 | 0.041 | 0.1904 | No |
| 25 | DLAT | dihydrolipoamide S-acetyltransferase [Source:HGNC Symbol;Acc:HGNC:2896] | 5647 | 0.020 | 0.1710 | No |
| 26 | ACSBG2 | acyl-CoA synthetase bubblegum family member 2 [Source:HGNC Symbol;Acc:HGNC:24174] | 5970 | -0.002 | 0.1460 | No |
| 27 | HACD2 | 3-hydroxyacyl-CoA dehydratase 2 [Source:HGNC Symbol;Acc:HGNC:9640] | 6162 | -0.017 | 0.1322 | No |
| 28 | ACSL6 | acyl-CoA synthetase long chain family member 6 [Source:HGNC Symbol;Acc:HGNC:16496] | 6505 | -0.043 | 0.1080 | No |
| 29 | SLC25A1 | solute carrier family 25 member 1 [Source:HGNC Symbol;Acc:HGNC:10979] | 6509 | -0.043 | 0.1102 | No |
| 30 | PDHX | pyruvate dehydrogenase complex component X [Source:HGNC Symbol;Acc:HGNC:21350] | 6760 | -0.060 | 0.0942 | No |
| 31 | SLC26A1 | solute carrier family 26 member 1 [Source:HGNC Symbol;Acc:HGNC:10993] | 7618 | -0.128 | 0.0348 | No |
| 32 | PDK3 | pyruvate dehydrogenase kinase 3 [Source:HGNC Symbol;Acc:HGNC:8811] | 7661 | -0.132 | 0.0391 | No |
| 33 | ELOVL2 | ELOVL fatty acid elongase 2 [Source:HGNC Symbol;Acc:HGNC:14416] | 8080 | -0.161 | 0.0158 | No |
| 34 | ACSL3 | acyl-CoA synthetase long chain family member 3 [Source:HGNC Symbol;Acc:HGNC:3570] | 8170 | -0.169 | 0.0185 | No |
| 35 | ACLY | ATP citrate lyase [Source:HGNC Symbol;Acc:HGNC:115] | 8173 | -0.169 | 0.0280 | No |
| 36 | GCDH | glutaryl-CoA dehydrogenase [Source:HGNC Symbol;Acc:HGNC:4189] | 8180 | -0.170 | 0.0372 | No |
| 37 | ELOVL1 | ELOVL fatty acid elongase 1 [Source:HGNC Symbol;Acc:HGNC:14418] | 8406 | -0.186 | 0.0303 | No |
| 38 | PDHA2 | pyruvate dehydrogenase E1 subunit alpha 2 [Source:HGNC Symbol;Acc:HGNC:8807] | 9124 | -0.241 | -0.0117 | No |
| 39 | ACSL5 | acyl-CoA synthetase long chain family member 5 [Source:HGNC Symbol;Acc:HGNC:16526] | 9136 | -0.243 | 0.0014 | No |
| 40 | ACACA | acetyl-CoA carboxylase alpha [Source:HGNC Symbol;Acc:HGNC:84] | 9177 | -0.246 | 0.0123 | No |
| 41 | PANK3 | pantothenate kinase 3 [Source:HGNC Symbol;Acc:HGNC:19365] | 9286 | -0.255 | 0.0185 | No |
| 42 | SLC26A2 | solute carrier family 26 member 2 [Source:HGNC Symbol;Acc:HGNC:10994] | 9523 | -0.276 | 0.0159 | No |
| 43 | PPT2 | palmitoyl-protein thioesterase 2 [Source:HGNC Symbol;Acc:HGNC:9326] | 10103 | -0.331 | -0.0102 | No |
| 44 | ACSL1 | acyl-CoA synthetase long chain family member 1 [Source:HGNC Symbol;Acc:HGNC:3569] | 10705 | -0.394 | -0.0345 | No |
| 45 | ACACB | acetyl-CoA carboxylase beta [Source:HGNC Symbol;Acc:HGNC:85] | 10955 | -0.425 | -0.0296 | No |
| 46 | ELOVL5 | ELOVL fatty acid elongase 5 [Source:HGNC Symbol;Acc:HGNC:21308] | 11272 | -0.467 | -0.0275 | No |
| 47 | ACSL4 | acyl-CoA synthetase long chain family member 4 [Source:HGNC Symbol;Acc:HGNC:3571] | 11327 | -0.475 | -0.0045 | No |
| 48 | PDK4 | pyruvate dehydrogenase kinase 4 [Source:HGNC Symbol;Acc:HGNC:8812] | 11384 | -0.483 | 0.0187 | No |
| 49 | SLC25A16 | solute carrier family 25 member 16 [Source:HGNC Symbol;Acc:HGNC:10986] | 11411 | -0.488 | 0.0446 | No |
| 50 | DCAKD | dephospho-CoA kinase domain containing [Source:HGNC Symbol;Acc:HGNC:26238] | 11650 | -0.525 | 0.0561 | No |
| 51 | DIP2A | disco interacting protein 2 homolog A [Source:HGNC Symbol;Acc:HGNC:17217] | 12433 | -0.719 | 0.0363 | No |
Table: GSEA details [plain text format]

  

Fig 2: GOBP\_NUCLEOSIDE\_BISPHOSPHATE\_BIOSYNTHETIC\_PROCESS      
 Blue-Pink O' Gram in the Space of the Analyzed GeneSet

  

Fig 3: GOBP\_NUCLEOSIDE\_BISPHOSPHATE\_BIOSYNTHETIC\_PROCESS: Random ES distribution      
 Gene set null distribution of ES for **GOBP\_NUCLEOSIDE\_BISPHOSPHATE\_BIOSYNTHETIC\_PROCESS**
